# How to Magnetize

**Published:** 1891-09

**Authors:** 


### How to Magnetize; or Mesmerism and Clairvoyance, a Practical Treatise on the Choice, Management, and Capabilities of Subjects, with Instructions on the Manner of Procedure, by James Victor Wilson. Price, 25 cents.

The benefits of mesmerism and arguments in favor of it are given with rules for the selection of good subjects, and the processes explained, with miscellaneous observations. Somnambulism and Clairvoyance are defined, counsels and cautions, with advice to subjects, are found, and the value of mesmerism as a curative, and an aid to physicians, is quite fully considered. The work closes with a valuable chapter on Animal Magnetism as a therapeutic means, written by Dr. Fleming, and read before the Medical Society of the County of New York, in which the accounts of remarkable cases are given, and references to eminent authority intended to show that animal magnetism is an established fact.

This may be read with profit by every one, whether specially interested in the subject or not. It will be sent, postpaid, on receipt of price in stamps, 25 cents. Address Fowler & Wells Co., No. 775 Broadway, N. Y.

